# Mushroom-Derived Medicine? Preclinical Studies Suggest Potential Benefits of Ergothioneine for Cardiometabolic Health

**DOI:** 10.3390/ijms22063246

**Published:** 2021-03-23

**Authors:** Daniel Lam-Sidun, Kia M. Peters, Nica M. Borradaile

**Affiliations:** Department of Physiology and Pharmacology, Schulich School of Medicine and Dentistry, Western University, London, ON N6A 5C1, Canada; dlamsidu@uwo.ca (D.L.-S.); kpeter44@uwo.ca (K.M.P.)

**Keywords:** mushrooms, ergothioneine, antioxidant, anti-inflammatory, type 2 diabetes, metabolic syndrome, cardiovascular disease

## Abstract

Medicinal use of mushrooms has been documented since ancient times, and in the modern world, mushrooms have a longstanding history of use in Eastern medicine. Recent interest in plant-based diets in Westernized countries has brought increasing attention to the use of mushrooms and mushroom-derived compounds in the prevention and treatment of chronic diseases. Edible mushrooms are the most abundant food sources of the modified amino acid, ergothioneine. This compound has been shown to accumulate in almost all cells and tissues, but preferentially in those exposed to oxidative stress and injury. The demonstrated cytoprotectant effect of ergothioneine has led many to suggest a potential therapeutic role for this compound in chronic conditions that involve ongoing oxidative stress and inflammation, including cardiovascular and metabolic diseases. However, the in vivo effects of ergothioneine and its underlying therapeutic mechanisms in the whole organism are not as clear. Moreover, there are no well-defined, clinical prevention and intervention trials of ergothioneine in chronic disease. This review highlights the cellular and molecular mechanisms of action of ergothioneine and its potential as a Traditional, Complementary and Alternative Medicine for the promotion of cardiometabolic health and the management of the most common manifestations of cardiometabolic disease.

## 1. Introduction

Over the last decade, the naturally occurring modified amino acid, L-ergothioneine (EGT), has gained much attention as a potential therapeutic compound [1,2,3,4]. EGT is a unique thiol derivative of histidine and was first discovered by Charles Tanret in 1909 in the ergot fungus, *Claviceps purpurea* [5]. Although EGT is widely distributed within tissues of both plants and animals, it is exclusively synthesized in non-yeast fungi, actinomycete bacteria, lactobacillus bacteria, and some cyanobacteria [6,7,8,9]. Plants absorb EGT produced by microorganisms in the soil via their roots, whereas animals and humans acquire EGT only through their diet [10]. Edible mushrooms are the food source most enriched in EGT, while other dietary sources include some meat products, oat bran and beans [10]. EGT has been shown to accumulate in almost all cells and tissues, but preferentially in those exposed to oxidative stress and injury [1]. In fact, many reports have demonstrated a cytoprotectant effect of EGT in vitro and have suggested a potential therapeutic role for this compound in various conditions including cardiovascular diseases, chronic inflammatory diseases, UV damage, neuronal injury, eye disorders, kidney diseases, cancer and cellular aging [1,2,3,4]. However, the in vivo and clinical effects of EGT in chronic disease and its underlying mechanisms of action are not well-defined. Herein we review the potential beneficial properties of EGT and its possible utility as a Traditional, Complementary and Alternative Medicine (TCAM) in the management of the most common manifestations of cardiometabolic disease, including type 2 diabetes mellitus, cardiovascular disease, metabolic syndrome, and non-alcoholic fatty liver disease (NAFLD).

## 2. Mushrooms as Medicine

### 2.1. Traditional Use of Mushrooms

The medicinal use of mushrooms has been documented by ancient civilizations, and in the modern world, medicinal mushrooms have a longstanding history of use in Asian countries [11]. Within the past decade, interest in plant-based diets in Westernized countries has brought more widespread attention to the role of mushrooms in the prevention and treatment of chronic diseases [12]. There are approximately 12,000 species of mushrooms worldwide, of which about 2000 are edible. Current estimates suggest that nearly 200 wild species are used therapeutically [13]. Mushrooms contain an abundance of potential nutraceutical compounds including polysaccharides (β-glucans), vitamins, terpenes, ergosterol and polyphenols [14]. However, much recent interest has focused on EGT, which is particularly enriched in and considered relatively unique to mushrooms [3].

### 2.2. Mushrooms as a Source of EGT

EGT is ubiquitously present in almost all cells and tissues of both plants and animals. Plants are able to absorb EGT through their roots via a symbiotic relationship with fungi present in soil [10]. The concept that animals absorb EGT solely from the diet was initially recognized through a study of grain-fed versus casein-fed pigs, which showed that only grain-fed pigs had detectable concentrations of EGT in the blood [15]. In the early 2000s, it was established that mushrooms are the most abundant dietary source of EGT, with concentrations ranging between 0.21 and 2.6 mg/g of dry weight depending on mushroom species [16]. Edible mushrooms of the Basidiomycota division are able to synthesize EGT, unlike higher order plants and animals [6]. Mushrooms of the genus *Pleurotus*, commonly known as oyster mushrooms, appear to contain the highest amount of EGT, whereas white button mushrooms *Agaricus bisporus* contain lower concentrations but are also consumed in larger quantities. A typical serving of whole mushrooms can provide between 1 and 26 mg of EGT [16]. Recent estimates of EGT intake in general populations of European countries and the United States suggest averages between 0.051–0.255 mg EGT/kg bw/day, with Italy having the highest consumption [17]. Several groups have demonstrated the bioavailability of EGT from mushroom sources [18,19,20]. Daily consumption of 100 g of white button mushrooms for four months resulted in significantly increased EGT concentration in the blood [21]. Furthermore, consumption of a single dose of 16 g of mushroom powder has been reported to increase EGT concentration in red blood cells [18]. Therefore, mushrooms and mushroom powders represent a unique food source of bioavailable EGT.

### 2.3. Synthetic EGT for Supplemental Use

The discovery that EGT can accumulate in tissues upon mushroom consumption sparked interest in the development of a sustainable supply of synthetic EGT and similar biomimetics. The synthesis of enantiomerically pure L-(+)-EGT was first described by Xu and Yadan in 1995 [22]. Since then, nature-identical biomimetics of EGT have been produced for commercial use (Mironova Labs Inc., Tetrahedron, Blue California). Recently, synthetic EGT marketed as Ergoneine® (Tetrahedron) was approved as a safe, novel supplement with a recommended daily dose of 30 mg/day for adults and 20 mg/day for children by the European Food Safety Authority [23], and ErgoActive (Blue California) was recognized as safe by the Food and Drug Administration in the United States [24]. Although the absence of toxicity at high millimolar concentrations has been shown in rodent models [25], studies in human subjects evaluating the safety and efficacy of EGT supplementation are limited.

## 3. EGT Pharmacokinetics

### 3.1. EGT Transport

The plasma membrane is impermeable to EGT and uptake is dependent on the presence of organic cation transporter novel type-1 (OCTN-1) encoded by the *SLC22A4* gene [26]. The significance of OCTN-1-mediated transport of EGT into various tissues in health and disease has been recently reviewed [3,4]. OCTN-1 is a highly selective Na^+^- and pH-dependent transporter of EGT, which has low selectivity for other compounds, including the structurally similar intermediate product, hercynine [27]. Silencing of *SLC224A* has been shown to inhibit EGT uptake and whole-body knock-out (KO) of OCTN-1 in mice was associated with complete absence of EGT in both blood and tissue, confirming an absence of alternative EGT uptake mechanisms [28].

### 3.2. Tissue Accumulation and Distribution of EGT

In animals, EGT has a wide tissue distribution, with tissue concentrations ranging from 100 µM–2 mM [2]. EGT is most abundant in the liver, erythrocytes, kidney, intestines, eye, bone marrow, seminal fluid, and lens of the eyes [29]. Interestingly, EGT tends to accumulate in tissues exposed to oxidative stress [1] and tissues at risk in some human diseases including fatty and fibrotic livers and infarcted hearts [30,31], leading Halliwell et al. to suggest that EGT is an “adaptive” antioxidant [3].

Many studies have described the accumulation of EGT from the diet [2,18,21], however, there are fewer studies characterizing EGT accumulation following administration of the pure compound. Cheah et al. were the first to report that following administration of pure EGT to humans, plasma EGT levels significantly increased during the administration period in a dose-dependent manner [20]. In mice, basal EGT concentrations are highest in liver, followed by whole blood, spleen, kidney, lung, heart, small intestine, eye, large intestine then brain. After seven days of EGT supplementation, most mouse tissues exhibited increased EGT content. Interestingly, however, after one day of EGT supplementation, only the liver and large intestine showed increased EGT content, suggesting that accumulation occurs more rapidly in the liver than in whole blood [29]. Similar results were found using a mouse model of chronic kidney disease, where the liver retained EGT levels despite impaired intestinal absorption [32]. EGT has also been shown to accumulate in guinea pig liver [30].

### 3.3. Metabolism and Excretion of EGT

Little is known about EGT metabolism in vivo, however, some studies have suggested three main by-products of EGT metabolism—hercynine, EGT-sulfonate and S-methyl EGT [29]. Hercynine appears to be the predominant metabolite of EGT, and it may be the most important metabolite in vivo [20,29]. Unlike many diet-derived polyphenols and anti-oxidants, which are rapidly metabolized and excreted, EGT appears to be highly retained, with relatively slow metabolic clearance [3,33]. Most recently, the high retention and slow excretion rate of EGT was demonstrated in humans following an oral dose of pure EGT [20]. These pharmacokinetic characteristics have been attributed to renal reabsorption of EGT [28,29].

## 4. Relevance to Cardiometabolic Disease

### 4.1. General Antioxidant and Anti-Inflammatory Effects

Numerous in vitro studies have demonstrated the ability of EGT to act as a general cytoprotectant with its various abilities to scavenge reactive oxygen and nitrogen species (ROS, RNS), protect against UV and gamma radiation-induced damage, chelate metal cations (Cu^2+^, Fe^2+^), and elicit anti-inflammatory effects. EGT is well known for its potential antioxidant capabilities as it scavenges hydroxyl radicals, singlet oxygen, hypochlorous acid, lipid peroxides, peroxynitrite and superoxide ions (one of the most harmful derivatives of molecular oxygen for the vascular endothelium). These activities have been extensively reviewed elsewhere [3,4,34]. Interestingly, EGT is more effective at scavenging peroxynitrite and singlet oxygen than glutathione [35,36]. Moreover, EGT accumulates intracellularly within mitochondria and nuclei to confer protection against oxidative DNA damage [1]. Animal studies have suggested that EGT may only play a role in tissues and cells that express high levels of oxidative stress, leaving basal ROS levels unchanged in healthy tissues [3]. Studies in cell cultures have shown that EGT can decrease pro-inflammatory cytokines IL-6, IL-1β and TNF-α [37,38]. However, fewer studies exist that elucidate the potential anti-inflammatory effects of EGT in vivo. Taken together, this evidence suggests that raising tissue levels of EGT may be beneficial in conditions involving chronic oxidative stress and inflammation. These conditions are characteristic of cardiometabolic disease that encompasses any or all of type 2 diabetes mellitus, cardiovascular disease, and NAFLD [39].

### 4.2. Type 2 Diabetes Mellitus

Type 2 diabetes mellitus causes significant complications in the cardiovascular and nervous systems through increased tissue oxidative stress and inflammation. This devastating metabolic disease arises when insulin-sensitive tissues such as the liver, muscle and adipose tissue become insulin resistant, eventually leading to pancreatic beta-cell failure, reduced insulin secretion, and ultimately abnormally high blood glucose levels [40]. Given that obesity and diet play critical roles in the development and control of type 2 diabetes, it may be beneficial for individuals either at risk of, or with overt type 2 diabetes to incorporate EGT-rich mushrooms into their diets in conjunction with healthy eating behaviors.

White button mushroom comprises 35–45% of total worldwide edible mushroom consumption and is widely cultivated throughout Europe and North America [41]. In a retrospective study of adults with pre-diabetes and two or more confirmed metabolic syndrome criteria, daily consumption of a standard serving of white button mushrooms (100 g, ~3.2 mg of EGT) for 16 weeks was associated with decreased systemic oxidative stress and inflammation. At the end of the 16-week dietary intervention, serum EGT concentrations were increased 2-fold compared to baseline, which was associated with a decrease in markers of oxidative stress and inflammation. Specifically, serum advanced glycation end products (AGE) (carboxymethyl lysine, methylglyoxal derivatives) were reduced, while increases were observed in oxygen radical absorbance capacity, an indicator of antioxidant response, and in adiponectin, an anti-inflammatory hormone [21].

Elevated levels of free fatty acids have been shown to initiate inflammation, leading to insulin resistance in skeletal muscle, a hallmark of type 2 diabetes. Studies using mouse C2C12 myoblasts suggest that EGT may protect against palmitic acid-induced cell death by decreasing p38 and c-Jun N-terminal kinases (JNK) phosphorylation, resulting in downregulation of the pro-inflammatory cytokine, IL-6. Given the well-established link between IL-6 and insulin resistance, this suggests that IL-6 may be an EGT target for preventing insulin resistance in skeletal muscle [37].

Vascular disease is a common complication in individuals with diabetes and several in vitro studies have indicated that EGT may protect the endothelium against glucose-induced oxidative stress. In endothelial cells (EC) treated with high glucose, pyrogallol, xanthine oxidase plus xanthine, hydrogen peroxide, and paraquat dichloride (all known inducers of oxidative stress), EGT decreased ROS production, improved cellular redox status, and increased cell viability [34,42]. EGT has also been shown to attenuate the reduction of acetylcholine-induced vasodilation in isolated rat arteries exposed to a variety of inducers of oxidative stress, and to improve the vasoresponsiveness of isolated basilar arteries from streptozotocin-induced diabetic rats [42]. Furthermore, EGT accumulates in EC through OCTN-1 but is quickly depleted after treatment with OCTN-1 siRNA. In EC, the protective effects of EGT against pyrogallol were abolished after OCTN-1 siRNA treatment, clearly indicating that cellular uptake and accumulation of EGT is required. The antioxidant and cytoprotective effects of EGT could be attributed to its ability to directly scavenge ROS, downregulation of the ROS producing enzyme, NADPH oxidase 1, or the induction of antioxidant enzymes, including glutathione reductase, catalase and superoxide dismutase [42]. D’Onofrio et al. reported similar findings, that EGT protected against high glucose-induced ROS production, cell senescence, and reduced cell viability. Furthermore, observations of EC cytotoxicity in conjunction with reduced EGT content in EC during high glucose exposure support the possibility of an important role for EGT in protecting against EC cytotoxicity during hyperglycemia. The mechanism whereby EGT protects against glucose-induced EC senescence may involve upregulation of sirtuins 1 and 6, which act to downregulate the adaptor protein p66Shc and the pro-inflammatory transcription factor NF-κB, respectively [43]. Sirtuins are a family of histone deacetylases responsible for the control of cellular metabolism and stress responses linked to cellular lifespan [44,45]. Sirtuin 1 has been shown to regulate eNOS, leading to decreased hyperglycemia-induced endothelial dysfunction [43,46]. Moreover, sirtuin 6 deficiency leads to increased expression of the pro-inflammatory cytokine IL-1β and IL-6 [47]. Overall, these studies in cultured EC and isolated small vessels suggest that EGT may be an effective modulator of cellular oxidative stress, inflammatory, and survival pathways in vascular cells.

In addition to its potential antioxidant and pro-survival effects in vascular cells and tissues exposed to high glucose, EGT may protect against reproductive defects in diabetes. In rat PC12 cells, a model of neuronal cells, EGT protected against glucose plus methylglyoxal-induced protein carbonylation, ROS production, and cell death. Moreover, EGT reduced the levels of AGE and its receptor (RAGE), and suppressed the expression of nuclear NF-κB, thus preventing mitochondria-associated apoptosis [48]. But perhaps more convincingly, studies in streptozotocin-induced diabetic pregnant mice, EGT protected against the development of neural tube defects and therefore reduced the incidence of embryo malformations. These effects were attributed to its antioxidant capabilities as there were no changes in maternal plasma glucose concentrations [49].

### 4.3. Cardiovascular Disease

Cardiovascular disease refers to disorders of the heart and blood vessels including coronary artery disease, heart failure, peripheral artery disease, and stroke [50]. In a recent population-based prospective study, Smith et al. proposed that identifying plasma metabolites associated with health-conscious food patterns could reveal specific metabolites that help to predict cardiometabolic disease and mortality. Using liquid chromatography-mass spectrometry, 112 plasma metabolites were identified in more than 3000 participants. During a median follow-up time of 21 years, EGT had the strongest association to health-conscious food patterns and was an independent predictor for lower risk of coronary artery disease, strokes, cardiovascular mortality, and overall mortality [51]. Although these findings do not indicate causality, the mechanisms underlying the potential benefit of EGT consumption in improving cardiovascular disease risk may be related to modulation of atherosclerosis development and progression. Atherosclerosis, the condition of fatty lesion (plaque) formation in the intima of arteries, is the underlying cause of most cardiovascular disease. Plaque formation begins with the accumulation of low-density lipoproteins (LDL) in the sub-endothelial space. Subsequent oxidation of LDL results in EC activation whereby adhesion molecules are expressed at the luminal surface of the endothelium, facilitating monocyte binding and migration into the sub-endothelial space. Within the sub-endothelial space, monocytes differentiate into macrophages and phagocytose oxidized LDL particles to become foam cells, a cellular hallmark of early atherosclerotic plaques [52].

Several studies have suggested that EGT-containing mixtures and EGT may affect the initial stages of atherosclerosis by mitigating LDL oxidation, monocyte adhesion to EC, and processes that contribute to foam cell formation. Crude aqueous gray oyster mushroom extract containing EGT has been shown to protect against hydrogen peroxide-induced human aortic EC death. Moreover, this mushroom extract reduced the formation of conjugated dienes and thiobarbituric acid reactive substances (TBARS) in EC, which are involved in the initial and late stages of human LDL oxidation, respectively [53]. Although preliminary, studies determining the bioavailability of EGT in humans from brown cremini mushroom powder showed a trend toward reducing the postprandial triglyceride response [18]. Postprandial triglycerides are known to increase the risk of coronary artery disease by contributing to the formation of foam cells. Furthermore, IL-1β-induced expression of the cell surface adhesion molecules V-CAM1, I-CAM1 and E-Selectin was suppressed by EGT in human aortic EC. Consequently, IL-1β-stimulated binding of human monocytes to EC was reduced in co-culture experiments [54].

Beyond its potential effects on atherosclerosis, EGT may modulate the development and progression of cardiovascular disease through direct effects on vascular and heart function. Studies by Gokce et al. suggested a role for EGT in promoting aortic relaxation [55]. This appeared to be endothelium-dependent, as the effect was abolished in denuded aortic ring preparations or by pre-treatment with the nitric oxide (NO) synthase inhibitor, L-NAME. Pre-treatment with EGT did not affect acetylcholine-induced relaxation in either intact or endothelium-denuded preparations, suggesting that EGT does not interfere with basal or agonist-stimulated production of NO. Follow-up studies indicated that EGT prevents the reduction of acetylcholine-induced relaxation of aortic rings caused by diethyldithiocarbamate and hypoxanthine plus xanthine oxidase, both potent inducers of superoxide formation [55]. In vivo, EGT concentrations were found to be elevated in the hearts of mice after permanent coronary ligation-induced heart failure and pressure overloaded-induced cardiac hypertrophy, conditions that increased oxidative stress and insulin resistance. This suggests that the accumulation of EGT observed may serve a protective function in this setting [31]. Furthermore, earlier studies by Arduini et al. in Langendorff rat heart preparations suggested that EGT protects against ischemia-induced myocardial damage, as indicated by reduced lactate dehydrogenase release. Corresponding in vitro assays suggested that the reduction of ferrylmyoglobin by EGT might be the underlying mechanism for decreased ischemic myocardial damage [56]. In light of these observations in aorta and heart tissues, further studies of the effects of EGT on large vessel and myocardial function under the metabolic stresses associated with obesity and type 2 diabetes are warranted.

### 4.4. Metabolic Syndrome and NAFLD

Non-alcoholic fatty liver disease (NAFLD) is common in individuals with obesity, metabolic syndrome, and type 2 diabetes, and may be independently associated with the development of atherosclerosis [57]. NAFLD is progressive and chronic, beginning with simple steatosis. Over time, steatosis and continued exposure to incoming lipid can result in non-alcoholic steatohepatitis (NASH) which is characterized by liver oxidative stress, inflammation, and fibrosis. End stages of NAFLD include cirrhosis and hepatocellular carcinoma [58]. Studies investigating the roles of EGT in the liver are limited but show promise in protecting against NAFLD.

Studies in larger rodent models suggest a liver-protective effect for EGT under various conditions. In a high cholesterol-fed guinea pig model of NAFLD, EGT accumulation in the liver significantly correlated with increased cholesterol and iron levels [30]. Elevated levels of iron have been previously associated with NAFLD, and may promote liver damage through the generation of ROS via Fenton chemistry [59]. Interestingly, iron depletion and iron chelators have benefit in NAFLD [60], suggesting that EGT accumulation observed in the guinea pig model may be a defensive mechanism initiated by the liver as it is a known metal chelator [1]. In support of this, expression of the EGT transporter, OCTN-1, and its regulatory transcription factor, RUNX1, were found to be upregulated in this model. Notably, despite advanced hepatic steatosis and liver damage (indicated by increased plasma alanine transaminase and gamma-glutamyl transpeptidase levels), there were no significant increases in hepatic lipid peroxidation and oxidative protein damage. Possible explanations for this observation include ROS scavenging, iron chelation or the upregulation of heat shock protein 70 (HSP70) by EGT [30]. Induction of HSP70 has been shown to protect hepatocytes against ischemia-reperfusion injury [61] and hepatotoxic compounds such as concanavalin A [62] and carbon tetrachloride [63]. In further support of this concept, administration of EGT protected rat livers against ferric-nitrilotriacetate-induced oxidative damage and ischemia-reperfusion-induced lipid peroxidation, in association with increased expression of HSP70. Liver damage was reduced, and survival rates were increased by 30% in these animals [61].

Recent studies in mice by Tang et al. also showed promising results for the effects of EGT on NAFLD [64]. Animals fed an atherogenic high fat diet to induce NASH showed increased expression of hepatic OCTN-1 in association with liver fibrosis. Moreover, OCTN-1 KO mice treated with the hepatotoxins, dimethylnitrosamine (DMN) or concanavalin A, to induce liver fibrosis showed increased fibrotic tissue, activated hepatic stellate cells (HSC), Kupffer cells, oxidative stress and lipid peroxidation compared to wild-type mice. Further investigation in wild-type mice treated with DMN showed increased liver expression of OCTN-1, EGT accumulation, and colocalization of OCTN-1 with HSC suggesting that EGT plays a specific role in HSCs and not in hepatic parenchymal cells. In support of this, in vitro assays using human HSC line LI90 showed functional expression of OCTN-1 via time-dependent EGT uptake that was reduced by OCTN-1 siRNA treatment [64]. In addition, OCTN-1 gene expression and EGT accumulation was induced in activated HSC following treatment with the fibrosis-related cytokine, TNF-α. Thus both in vivo and in vitro evidence from the work of Tang et al. [64] suggests that upon activation of HSCs during fibrosis, OCTN-1 upregulation and subsequent EGT accumulation may act as a defensive mechanism against the progression of liver fibrosis. Furthermore, oral administration of EGT can suppress oxidative stress and liver fibrosis in mice treated with DMN, supporting the potential use of EGT as a dietary hepatoprotective supplement. Similar to the studies described in the previous paragraph, a proposed mechanism for the beneficial effect of EGT in HSC may involve protection against oxidative stress by acting directly as a ROS scavenger or indirectly as a genomic regulator. Support for the latter comes from studies showing increased NADPH oxidase 4 gene expression in OCTN-1 KO mice and decreased expression in LI90 cells treated with EGT [64]. NADPH oxidase 4 generates ROS and could be involved in the progression of liver fibrosis, as it is highly expressed in both hepatocytes and HSC [64]. Interestingly, accumulation of EGT in the liver is also thought to occur in sinusoidal EC, [65] which, like HSC, are a non-parenchymal cell type involved in the coordinated progression of NAFLD and atherosclerosis [57].

## 5. Conclusions

**Figure 1:**
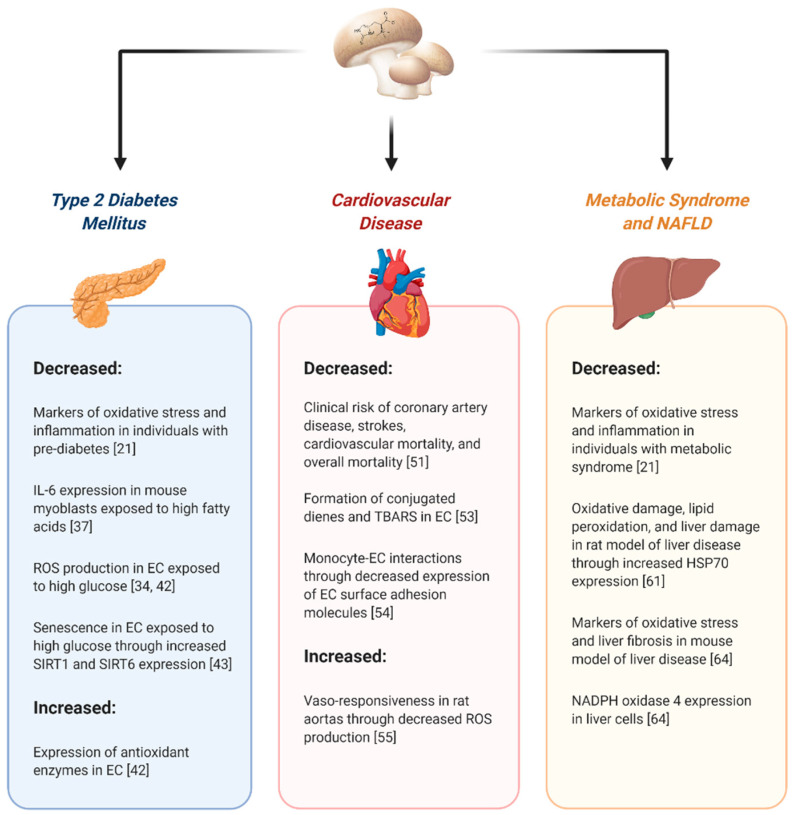
Potential cardiometabolic benefits of ergothioneine (EGT) consumption, supplementation, or treatment. Increased dietary consumption, supplementation, or experimental treatments with EGT have been associated with potentially beneficial effects in cell culture, small animal, and retrospective studies in human subjects. ROS, reactive oxygen species; EC, endothelial cells; IL-6, interleukin-6; SIRT, sirtuin; TBARS, thiobarbituric acid reactive substances; HSP70; heat shock protein 70.

Increased dietary consumption, dietary supplementation, and experimental treatments with EGT have been associated with beneficial effects in cell cultures and in small animal models with manifestations of cardiometabolic disease—type 2 diabetes mellitus, cardiovascular disease, metabolic syndrome and NAFLD. Retrospective studies in humans have identified associations between EGT consumption, blood EGT concentrations, and improvements in markers of oxidative stress and inflammation [21], and lower risk of cardiometabolic disease and mortality [51]. Under conditions associated with these chronic diseases, the potential therapeutic effects of EGT appear to be primarily related to its antioxidant characteristics. Furthermore, under conditions of dysfunctional metabolism associated with type 2 diabetes and metabolic syndrome, EGT appears to be anti-inflammatory (Figure 1). However, it must be recognized that to date there is no direct, prospective evidence linking mushroom or mushroom product consumption, EGT intake, and cardiometabolic benefits in humans. There are also additional considerations for the use of any natural product in chronic disease management. Many have poor oral bioavailability, some can elicit adverse effects at high doses, and some can inhibit drug-metabolizing enzymes or drug transporters, therefore having the potential to affect drug disposition. Interestingly, due to its efficient uptake by a selective transporter (OCTN-1), its accumulation in tissues exposed to oxidative stress, and the apparent tolerance of tissues to high concentrations of this compound, EGT may hold more promise than other natural products. Given the recent safety approval of commercial EGT supplements in both Europe and North America, additional studies of the therapeutic mechanisms of action and prospective clinical trials of the effects of EGT in cardiometabolic disease are warranted.

## Data Availability

No new data were created or analyzed in this study. Data sharing is not applicable to this article.
